# An improved burden-test pipeline for identifying associations from rare germline and somatic variants

**DOI:** 10.1186/s12864-017-4133-4

**Published:** 2017-10-16

**Authors:** Yu Geng, Zhongmeng Zhao, Xuanping Zhang, Wenke Wang, Xingjian Cui, Kai Ye, Xiao Xiao, Jiayin Wang

**Affiliations:** 1 School of Electronic and Information Engineering, Xi’an Jiaotong University, Xi’an, 710049 Shaanxi China; 2 School of Management, Xi’an Jiaotong University, Xi’an, 710049 Shaanxi China; 3 Institute of Data Science and Information Quality, Shaanxi Engineering Research Center of Medical and Health Big Data, Xi’an Jiaotong University, Xi’an, 710049 Shaanxi China; 4 State Key Laboratory of Cancer Biology, Xijing Hospital of Digestive Diseases, Xi’an, 710032 Shaanxi China; 5 Jinzhou Medical University, Jinzhou, Liaoning, 121001 China

## Abstract

**Background:**

Identifying rare germline and somatic variants associated with cancer progression is an important research topic in cancer genomics. Although many approaches are proposed for rare variant association study, they are not fit for cancer sequencing data due to multiple issues, such as overly relying on pre-selection, losing sight of interacting hotspots, etc.

**Results:**

In this article, we propose an improved pipeline to identify germline variant and somatic mutation interactions influencing cancer susceptibility from pair-wise cancer sequencing data. The proposed pipeline, *RareProb-C* performs an algorithmic selection on the given variants by incorporating the variant allelic frequencies. The interactions among the variants are considered within the regions which are limited by a four-gamete test. Then it filters singular cases according to the posterior probability at each site. Finally, it outputs the selected candidates that pass a collapse test.

**Conclusions:**

We apply *RareProb-C* on a series of carefully constructed simulation cases and it outperforms six existing genetic model-free approaches. We also test *RareProb-C* on 429 TCGA ovarian cancer cases, and *RareProb-C* successfully identifies the known highlighted variants which are considered increasing disease susceptibilities.

## Background

Over recent decades, large cancer genome projects, such as TCGA and ICGC [1, 2], greatly promote the achievements on cancer genomics. One of the important research topics is to comprehensively identify the germline and somatic variants that contribute to cancer susceptibility. Several standard pipelines for detecting germline variants and somatic mutations have been developed. For each cancer patient, these pipelines require two sets of sequencing data, one of which is from tumor tissue, while the other collects from normal tissue. The variant calls from a pair of such two sets are compared. Germline variants are expected to be observed in both sets, while the plausible differences may represent genuine somatic mutations.

Deleterious germline variants inheritance are usually confined to a small population, whose minor allele frequencies are usually very low. Interacting with germline ones, highly recurrent somatic mutations only make up a small proportion of total somatic events. Low minor allele frequencies can terribly hurt the statistical power and odd ratio in association analysis. To tackle this issue, existing computational approaches for germline and somatic variants widely adopt the collapsing strategy, as known as the burden-test. It is a major technique for rare variant association study. The basic idea of collapsing strategy is to merge the given variants to one or multiple virtual loci, whose minor allele frequency(ies) is(are) obviously increased. The statistical tests are then applied to these virtual loci, together with common variants. For example, the mutations from the genes that are on the same pathway are often collapsed to a single virtual locus. The successful cases of using collapsing strategy have reported many rare variants that contribute to the susceptibility of a number of complex diseases and traits, including mutlipe types of cancer [3–6].

Any burden-test approaches could be classified into one of two categories: genetic model-based or genetic model-free. Genetic model-based approaches assume that the given variants are finely selected, while the model-free ones generate a set of candidate variants from given ones. Recent published approaches prefer the model-free strategy because model-free approaches are more aggressive in identifying novel deleterious events. The major diversity of the existing approaches is presented in the ways of selecting candidate variants. Some approaches weight the given variants before statistical tests, such as the rare variant weighted aggregated statistic (*RWAS*) [7], the likelihood ratio test (*LRT*) [8] and two weighted score tests with branching under ratios (*BUR*) and likelihood-based model branching (*LiMB*), respectively [9]. Regression is another idea to refine the given variants, such as the kernel-based association test (*KBAT*) [10], the sequence kernel association tests (*SKAT*) [11, 12] and convex-concave rare variant selection method (*CCRS*) [13]. *RareCover* is considered the first algorithmic selection approach, which filters the variants via a *χ* ^2^ aggregation greedy strategy [14]. Both *RareProb* [15] and the logistic Bayesian LASSO (*LBL*) [16] improve the selection strategy of *RareCover*, where *RareProb* implements a hidden Markov random field model and *LBL* incorporates a posterior Bayesian score calculated by a Markov chain framework.

Although different burden-test approaches vary in the means of collapsing, the “common disease, rare variant” hypothesis limits their applications in cancer genomics [17]. This hypothesis suggests that a set of independent variants with low MAFs and modest penetrances jointly affect a complex trait, where each may only explain a small portion of the phenotypes [5, 6]. Here, the interactions among rare variants are suggested to be either non-existent or too weak to be observed [7, 8, 14, 18]. However, it is reported that the germline variant and somatic mutation interactions influencing cancer susceptibility is involved in more than 3% of cancer cases across multiple cancer types [4]. For example, the two-hit hypothesis serves as a classic genetic model for the DNA repair and tumor suppressor genes [19]. Moreover, several computational models are proposed to capture such interactions. For example, the significant mutated gene test has successfully supported the research on the landscape of the interacting somatic mutations across 12 major cancer types [3]. Such interactions are also observed in clonal expansion analysis [20]. It is also reported that the somatic copy-number alternations may contribute to the potential selective advantages of the germline variants [4]. Second, tumor tissue is an admixture of clonal tumor cells and non-cancerous cells. And thus, sample contamination and clonal architecture are ineluctable. Furthermore, the variant allelic frequency at each site reveals differences among sub-groups of the given samples. Without considering these issues, a collapsing method would lose sensitivity and specificity, and the association approach could be weakened by decreasing the statistical powers.

We propose a burden-test pipeline in this article. This pipeline extends the existing *RareProb* framework proposed in [15] and the name of this pipeline is *RareProb-C*. *RareProb-C* is designed for cancer sequencing data, which considers the interactions among rare germline and somatic variants. To verify this pipeline, we conduct a series of simulation experiments with different settings, and compare *RareProb-C* to 6 popular approaches, which are 1) *RWAS*, *BUR* and *LiMB* from weighted-based group, 2) *CCRS* from regression-based group and 3) *RareCover* and *LBL* from algorithmic selection group. The results of *RareProb-C* show significant advantage in type-II error rates. *RareProb-C* is also tested on a set of 429 TCGA ovarian cancer cases. The association report provided by *RareProb-C* includes more highlighted variants, which are considered to be associated with increased cancer susceptibilities.

## Methods

*RareProb-C* consists of four components, and *RareProb* [15] becomes a core module of one component. The three new components are described in this section, whose functions are detecting interacting variants, estimating the significant mutated regions, and removing singular variants or cases. During implementation, all these components are executed simultaneously within a hidden Markov random field framework. Supposing that we are given *M* variants on a set of *N* samples. Variants could include both single-site ones and small indels. Large structural variations are not discussed here, as their functional analyses are often involved in either large LOH/CNV or more complex mechanisms [4]. Suppose the given *N* samples consist of $\frac {N}{2}$ cases and $\frac {N}{2}$ controls. If the number of cases is not equal to the number of controls, the following equations can be used simply by applying non-centrality parameters.

### Detecting interacting variants

For germline variant *s*, let *θ*_*s*_ denote the variant allelic frequency (VAF) in a tumor sample and *ρ*_*s*_ denote the variant allelic frequency in the paired normal sample. Let $c_{s}^{+}$ and $c_{s}^{-}$ represent the number of reads supporting the mutation at site *s* in the tumor and normal samples, respectively. Two binomial distributions can be drawn for the tumor and normal samples: $c_{s}^{+} \sim \text {Bin}\left (d_{s}^{+},\theta _{s}\right)$ and $c_{s}^{-} \sim \text {Bin}\left (d_{s}^{-},\rho _{s}\right)$, where $d_{s}^{+}$ and $d_{s}^{-}$ denote the respective read depths. For this site across samples, the statistic of the difference between *θ*_*s*_ and *ρ*_*s*_ is calculated by a pair-wise *t*-test. Similar to the linear kernel function [11], which calculates genetic similarities, we weight the interactions of two variants *s* and *s* ^′^ by $\omega _{s,s'} \sim \frac {2t_{s}t_{s'}}{t_{s}^{2} + t_{s'}^{2}}$, which affects how likely these two variants would be collapsed. For somatic mutations, only *θ*_·_ and $c_{\cdot }^{+}$ are considered. By applying clonal architecture analysis [3], *θ*_·_ are clustered into multiple sub-clones and the correlation coefficient $\phantom {\dot {i}\!}\omega _{\cdot,\cdot ^{\prime }}$ of each mutation-pair within a sub-clone is obtained simultaneously.

Furthermore, the interactions among multiple variants may vary from two adjacent sites to a few sites within a certain region/pathway, e.g., within a gene or multiple genes. Let *G*_·,*s*_ represent the genotypes at any site *s* across all given samples. Here we assume that the observed genotype reflects a Bayesian classifier from multiple factors, such as $c_{s}^{+}$ and $c_{s}^{-}$. Let *m*(*G*_·,*s*_) denote the classifier model for each case at site *s*. Then, the interactions among *s* and neighboring variants *n*(*s*) can be described as the conditional probability of *m*(*G*_·,*s*_) undergoing $\phantom {\dot {i}\!}m\left (G_{\cdot,s'}\right)$, where $\phantom {\dot {i}\!}s^{\prime }\in n(s)$. To clearly describe the patterns of interactions, we have

$${} P\left(G_{\cdot,s} | G_{\cdot,s_{n(s)}}\right) \propto \text{exp}\left(\tau m\left(G_{\cdot,s}\right) + \nu \sum_{s'\in n(s)} \omega_{s,s'} m\left(G_{\cdot,s'}\right) \right) $$

where the first component, *τ* *m*(*G*_·,*s*_) represents the solo effects from *s* itself, while the second component describes the effects from the interacting ones.

The variant allelic frequency for each site consists of an *N*-dimensional vector. Let the vector be a state in the Markov random field. Then, state *H*_*s*_=[*G*_·,*s*_]^−1^ corresponding to site *s*, and based on the Markov-Gibbs equivalence, for two interacting variants *s* and *s* ^′^, the interaction could be represented as a transition probability between *H*_*s*_ and $\phantom {\dot {i}\!}H_{s^{\prime }}$, which is

$$\begin{aligned} P\left(H_{s} | H_{s^{\prime}}, \theta \right) \propto& \text{exp}\left(\sum_{i=1}^{N} \mu + \left(1 - \mu\right) f\right.\\&\left. \left(-\theta d_{s,s' } + I_{s,s'}e^{-\theta d_{s,s'}}\right){\vphantom{\sum_{i=1}^{N}}}\right) \end{aligned} $$

where *μ* denotes the mutation rate by which a variant occurs independently, while *θ* denotes an unknown model parameter that describes the probability of a variant being located in a deleterious region. $\phantom {\dot {i}\!}d_{s,s^{\prime }}$ is the genetic distance between two adjacent sites *s* and *s* ^′^, which underlies the length of a region.

### Estimating the regions with interactions

The transition probability $\phantom {\dot {i}\!}P\left (H_{s} | H_{s^{\prime }}, \theta \right)$ models the interacting patterns between two sites, and we then bridge each interacting pattern to the region vector *R*. *R* can be shared across cases with similar features.

For any site *s*, vector *G*_·,*s*_ is used to estimate the status of region vector *R*_*s*_. To facilitate the computation, without loss of the Markov property, we first define two additional silent states affiliated to the state *H*_*s*_, which are $R_{s}^{e}$ and $R_{s}^{b}$. Then, we define that a Markov chain via state $R_{s}^{e}$ denotes that site *s* is in an elevated region; otherwise, the chain though $R_{s}^{b}$ denotes that site *s* is in a background region. Let $P_{s}^{e} = P\left (R = 1 | H_{s}\right)$ be the transition probability of site *s* within an elevated region, while $P_{s}^{b} = P\left (R = 1 | H_{s}\right)$ denotes the probability of site *s* within a background region and the Markov property limits $P_{s}^{e} + P_{s}^{b} = 1$. Thus, the probability of site *s* being located in an elevated region can be represented by

$${} p\left(R_{s}^{e}|H_{s},H_{s^{\prime}},R_{n(s)}\right) \propto \text{exp}\left(\tau B\left(P_{s}^{e},P_{s}^{b}\right) + \nu \sum_{s' \in n(s)} \omega_{s,s'} R_{s'}\right) $$

and the joint probability of latent vector *R* can be represented by $p\left (R;\Phi _{R}\right) \propto \text {exp}\left (\tau \sum _{s}^{M} R_{s} + \nu \sum _{s,s'} \omega _{s,s'} R_{s'}\right)$, where *s* ^′^∈*n*(*s*) and *Φ*_*R*_=(*τ*,*ν*). *τ* and *ν* are two model parameters balancing the importance of interactions, where *τ*+*ν*=1. $\phantom {\dot {i}\!}R_{s'} = P\left (R_{s'}|X\right)$, where *X* is a *M*-dimensional vector that each element represent the causal/non-causal status of the corresponding variant.

Furthermore, we require each region to pass the four-gamete test, which limits the length of a region and potentially reduces false positives. We adopt a maximal-*K*-cover pipeline to divide $G_{\frac {N}{2}\times M}$ into multiple compatible intervals, as proposed in [21]. This pipeline consists of a series of scanning algorithms. First, it establishes two interval sets, *I*_*lr*_ and *I*_*rl*_, and obtains intervals from the left-to-right algorithm and right-to-left algorithm, respectively, where each set contains *K* intervals. Second, a merging algorithm combines these 2*K* intervals to extract the overlapping genotypes with the same index, for example, *I*_*l*,*i*_ and *I*_*r*,*i*_. It then defines *K* cores according to the overlapping, denoted by *C*={*o*_1_,*o*_2_,⋯,*o*_*K*_}. Third, an uber-scan algorithm calculates *K*+*m* candidate intervals *U*={*u*_1_,*u*_2_,⋯,*u*_*K*_} and assigns these *K*+*m* intervals to each core in *C*. If *o*_*i*_ contains *u*_*j*_, then *u*_*j*_ is allocated in the *i*-th group. Finally, the maximal intervals are estimated via a dynamic programming algorithm. We restrict any region within one compatible interval, where the genotypes are restricted by having limited diversity.

### Estimating model parameters

Based on the Gibbs-Markov equivalence, a pseudo-likelihood iteration algorithm can solve this model to estimate the unknown model parameters and the hidden states. Again, due to the computational complexity, we introduce another two silent field states to facilitate the computation process. For any site *s*, let $\vec {J}_{i,\cdot }^{s}$ permute from $\phantom {\dot {i}\!}\vec {G}_{\cdot,s^{\prime }}$, where the transition probability from $J_{i,\cdot }^{s}$ to $\vec {G}_{\cdot,s'}$ is $\propto \frac {1}{N-1}$; let $\vec {J}_{i}^{s}$ duplicate from the vector $\phantom {\dot {i}\!}G_{\cdot,s^{\prime }}$, where the transition probability from $\vec {J}_{i}^{s}$ to $\vec {G}_{\cdot,s'}$ is 1. The other component of $\phantom {\dot {i}\!}P\left (H_{s} | H_{s^{\prime }},\theta \right)$ can be divided into two separate transition probabilities, namely, $P\left (J_{i}^{s} | R_{s}\right) = f\left (-\theta d_{s,s'}\right) + e^{-\theta d_{s,s'}}$ and $ P\left (J_{i,\cdot }^{s} | R_{s}\right) = \frac {1}{N-1}f\left (-\theta d_{s,s'}\right)$, where *f*(*x*) is (1−*e* ^−*x*^)/*N*.

Now, for the entire hidden Markov random field model, we need to estimate three unknown model parameters: the mutation rate *μ*, the deleterious region location likelihood *θ* and the regional Bayesian classifier $P_{\cdot }^{e}$. $P_{\cdot }^{b}$ fully depends on $P_{\cdot }^{e}$. The forward probability for the hidden state *R*_*s*_ is

$$\alpha_{R}(s) = \sum_{j=0}^{2N} \alpha_{R}\left(s - 1\right) P_{s}^{b}\cdot I + P_{s}^{e} \left(1 - I\right) P\left(R_{s}|X\right) $$

where the indicator function *I*=1 when *m* *o* *d* *e*(*j*,2)=0. The forward probability for $\vec {H}_{s}$ is

$$\alpha_{H}(s) = \sum_{j=0}^{N} \alpha_{H}\left(i,s-1\right)P\left(H_{s}\right) $$

Similarly, we have the forward and backward probabilities for hidden states *R*_*s*_ and *H*_*s*_ at the same site, which are *β*_*R*_(*s*) and *β*_*H*_(*s*).

We incorporate an EM algorithm described in [22] into the original iterated conditional mode algorithm in *RareProb* to estimate the model parameters and update the hidden states. In iteration *r*, the algorithm estimates the hidden parameters as follows:

$$\mu_{r+1} = \frac{\sum_{i=1}^{N}\sum_{j=0}^{M} I(H_{s} \neq G_{i,s})P(H_{s}|G,\mu_{r},\theta_{r})}{\sum_{i=1}^{N}\sum_{j=0}^{M} I\left(G_{i,s} > 0\right) P(H_{s} |G,\mu_{r},\theta_{r})} $$

and

$$\begin{aligned} \theta_{r+1} =& argmax \left(\sum_{i=1}^{M} P\left(J_{i}^{s} | R_{s}\right) \sum_{j=1}^{N} P\left(J_{i}^{s} | G,\mu_{r},\theta_{r}\right)\right. \\&\qquad \left.+ P\left(J_{i,\cdot}^{s} | G,\mu_{r},\theta_{r}\right) {\vphantom{ \sum_{i=1}^{M}}}\right) \end{aligned} $$

Let *ξ*_*R*_(*s*,*i*,*j*) represent the probability of *R*_*s*_ equal to *i* and $\phantom {\dot {i}\!}R_{s^{\prime }}$ equal to *j*, with the conditions of $\hat {R}$ and model parameters. Then, we have

$${} \xi_{R}\left(s,i,j\right) = \frac{\alpha_{R}(s,i) P\left(H_{i} | H_{j}\right) P\left(R_{s}|X\right) \beta_{R}(s,j)}{\sum_{i=1}^{2N} \sum_{j=1}^{2N} \alpha_{R}(s,i) P\left(H_{i} | H_{j}\right) P\left(R_{s}|X\right) \beta_{R}(s,j)} $$

thus,

$$\left(P_{s}^{b}\right)_{r+1} = \frac{\sum_{s}^{M} \xi_{R}\left(s,0,0\right)}{\frac{1}{N}\left(1 + \left(N - 1\right)e^{-\theta d_{s,s'}}\right)} $$

Once the differences in parameters between two iterations are less than a preset threshold(s), this algorithm terminates.

### Filtering singular cases

With the estimation of regions and causal/non-causal status for each variant, we also filter cases with different patterns of features via the posterior probability at each variant. We first bootstrap a set of samples from all of the cases and estimate the model structural parameters via the aforementioned algorithms. In the implementation, we include a short-cut to first filter out *L* samples with higher posterior probabilities, and then apply the bootstrap on those selected ones to speed up the process.The posterior probability at a specific site *s* carrying genotype *i* is

$$\zeta\left(G_{i,s}\right) \propto \text{exp}\frac{\alpha_{R}(s,i) \beta_{R}(s,j)}{\alpha_{R}(M,i)} $$

If the posterior probability *ζ*(*G*_*i*,*s*_) is less than $\zeta \left (\bar {G}_{i,s}\right)$, we consider the variant as one with different feature pattern. If a case carries a number of such variants, where the number is a user-setting parameter, we will eliminate it from downstream association analysis.

## Results and discussion

To test the performance of the proposed approach, we first apply *RareProb-C* to a series of simulated datasets under different configurations, and compare the results to 6 recent published methods. As mentioned above, according to the ways of selecting candidate variants, the existing model-free approaches could be summarized into one of three categories: weighted-based methods, regression-based methods and algorithmic selection methods. We choose at least one approach from each category. The approaches, to which we compare, include 1) *RWAS*, *BUR* and *LiMB* from weighted-based group, 2) *CCRS* from regression-based group and 3) *RareCover* and *LBL* from algorithmic selection group. We also apply *RareProb-C* to a set of 429 TCGA samples from the ovarian group, and then the results are verified by the literature review.

### Generating simulation datasets

We adopt the same way of generating simulation datasets as in [7, 8, 15]. It implements a fixed-number strategy. The number of causal variants, denoted by *C*, and the total number of variants *M* in each dataset are preset. To conduct a fair comparison, each rare variant in this experiment is generated independently because the existing approaches do not consider the interacting rare variants. In the real dataset later on, the interacting germline and somatic variants are involoved then. The assumption behind the fixed-number strategy is that linkage disequilibrium between rare variants does not exist. The minor allele frequency of each variant in these cases follows the Wright’s distribution:

$$f\left(\rho_{i}\right) \propto \left(\rho_{i}\right)^{\beta_{i} - 1}\left(1 - \rho_{i}\right)^{\beta_{L} - 1}e^{\sigma - \rho_{i}\sigma} $$

where *ρ*_*i*_ is the MAF at site *i*, and *σ* is the selection coefficient. A causal variant may be repaired to a neutral one with the probability of *β*_*L*_, while *β*_*i*_ is the probability of mutating a neutral variant to causal one. We adopt the same parameter setting as in [7, 8, 15], where we set *σ*=12.0, *β*_*i*_=0.001 and *β*_*L*_=0.00033. The relative risk (RR) of a causal variant is defined as $RR = \frac {\delta }{\left (1 - \delta \right)\rho _{i}} + 1$, where *δ* is the marginal population attributed risk (PAR), which is equal to the group PAR divided by the number of causal variants. The reason is that the sum of relative risk among all the variants should be equal to 1. The minor allele frequency in controls is controlled by relative risk, which is $\theta _{i} = \frac {RR \times \rho _{i}}{(RR - 1)\rho _{i} + 1}$. In each dataset, we first randomly choose *C* causal variants in a set of 100 variants and record the causal ones into *X*_*known*_. Then, according to the previous strategy, we generate 1000 cases and 1000 controls.

#### Comparisons between *RareProb-C* and the existing approaches

We first compare the statistical power and type-I error rate. For the statistical power, we take the same measurement used in [7, 8, 15], where the power of an approach is measured by the number of significant datasets among all the datasets with the same configuration, using a significance threshold of 2.5 ×10^−6^ based on the Bonferroni correction, assuming 20000 genes genome-wide. The type-I error rate of each experiment is defined as the probability of a preset causal variant not being selected for the set of candidate variants. We test exactly 100 datasets for each comparative experimental configuration, where the type-I error rates take the average. In this group of experiments, we hold the number of preset causal variants to 50 and vary the population attributed risk (PAR) from 0.02 to 0.05 and the results are shown in Table 1. According to Table 1, we are able to summarize that most of the approaches can achieve very high statistical powers, among which *RareProb-C* presents lower type-I error rates than the other approaches in most of the simulation configurations. We also would like to explain a little more for the two exceptions, *RareCover* and *LRT*. The type-I error rates of *RareCover* are calculated from the significant datasets only. Although *RareCover*’s rates are much lower than the other approaches, the statistical power is always the premise. For *LRT*, as there is no prior information for the simulation datasets to conduct variant selection, *LRT* involves all given variants into the likelihood ratio test. And thus, *LRT* does not have type-I error rates but always has 100% type-II error rates.

**Table 1:** The statistical powers and the type-I error rates of *RareProb-C* and other approaches on the simulation datasets

PAR	0.02		0.03		0.04		0.05	
Approach	Power	Type-I	Power	Type-I	Power	Type-I	Power	Type-I
RareProb-C	100%	34.22%	100%	25.06%	100%	22.33%	100%	24.33%
RareCover	71.67%	5.89%	62.67%	3.97%	54.55%	2.86%	47.73%	1.92%
LRT	99%	0%	100%	0%	100%	0%	100%	0%
LBL	100%	37.47%	100%	37.57%	100%	35.74%	100%	31.19%
BUR(0.95)	100%	55.03%	100%	60.16%	100%	64.85%	100%	59.06%
LiMB	100%	44.99%	100%	48.62%	100%	55.04%	100%	42.82%
CCRS	100%	27.82%	100%	43.49%	100%	56.86%	100%	67.73%

The population attributed risk (PAR) vaires from 0.02 to 0.05. The significance threshold is set to *P*<0.05

To further evaluate the performance, we also compute the type-II error rate. The type-II error rate of each experiment is defined as the probability of a preset neural variant being selected for the set of candidate variants. We test exactly 100 datasets for each comparative experimental configuration, where the type-II error rates take the average. In the following experiments, we vary the population attributed risk (PAR) from 0.02 to 0.05 and also enumerate the number of preset causal variants from 50 to 90 and the results are listed in Table 2. From Table 2 we can see that *RareProb-C* offers a significant improvement in reducing the type-II error rate, which is considered to be very important in clinical genomics.

**Table 2:** Comparison results among *RareProb-C* and the other 5 state-of-the-art approaches on the type-II error rates

PAR	Causal	Type-II error					
Approach		RareProb-C	LRT	LBL	BUR 0.95	LiMB	CCRS
0.02		12.89%	100%	32.99%	23.93%	42.78%	53.17%
0.03	50	15.21%	100%	30.09%	14.96%	57.37%	54.00%
0.04		17.28%	100%	19.78%	11.16%	47.99%	52.02%
0.05		20.90%	100%	25.51%	12.03%	52.76%	52.42%
0.02		12.20%	100%	26.06%	20.15%	56.10%	54.45%
0.03	60	17.05%	100%	26.18%	16.16%	60.48%	53.03%
0.04		20.75%	100%	39.02%	14.87%	65.71%	54.08%
0.05		23.29%	100%	31.14%	19.44%	58.82%	55.45%
0.02		12.29%	100%	31.88%	20.33%	67.80%	52.53%
0.03	70	19.10%	100%	33.11%	20.66%	67.94%	53.88%
0.04		22.39%	100%	33.32%	23.55%	71.77%	55.62%
0.05		22.51%	100%	32.09%	31.37%	75.55%	58.84%
0.02		11.39%	100%	32.66%	9.29%	80.07%	50.16%
0.03	80	17.85%	100%	48.38%	8.38%	76.71%	53.03%
0.04		22.98%	100%	38.21%	31.35%	84.29%	62.57%
0.05		15.57%	100%	42.83%	39.47%	78.66%	67.21%
0.02		11.79%	100%	36.91%	35.63%	91.26%	51.73%
0.03	90	20.42%	100%	40.49%	40.33%	89.29%	61.25%
0.04		21.28%	100%	39.91%	47.99%	91.26%	70.83%
0.05		14.72%	100%	48.87%	55.54%	88.79%	74.26%

The population attributed risk (PAR) still vaires from 0.02 to 0.05 and the number of preset causal variants enumerates from 50 to 90. The significance threshold is set to *P*<0.05

#### Null-model test for *RareProb-C*

We also apply a null-model test on *RareProb-C* to collect the dataset-level type-I error rate. The type-I error at dataset level measures how frequently a non-significant dataset (consisting of non-causal variants) is wrongly reported as a significant association dataset. We randomly generate one million datasets, each consisting of 1000 samples with 100 variants each. At each sample site, a mutation is assigned with the probability of 0.005. For each sample, it has the same probability of being set as a case or a control. Among these 10,000 datasets, *RareProb-C* only reports 9 significant datasets, which shows strong reliability. *LBL* reports 24 significant datasets. *CCRS* reports 122 significant datasets. Both *BUR* and *LiMB* report 0 significant datasets.

### Experiments on cancer sequencing data

We then apply *RareProb-C* to a real cancer sequencing dataset. This dataset consists of 429 TCGA serous ovarian cancer (OV) cases [1, 23]. Each case has one tumor sample with whole exome sequencing data and one normal sample with whole exome sequencing data. All of the data are aligned to human reference build 37 using BWA, and variants are identified using VarScan, GATK, and Pindel, with stringent downstream filtering to standardize specificity. Variant annotation is based on Ensembl release 70_37_v5. The variant list for association analysis contains 3050 germline truncation variants and 4724 somatic truncation mutations. Read count and variant allelic frequency analysis are performed by the bam-readcount tool, available at https://github.com/genome/bam-readcount. Somatic variants with VAFs can be downloaded from the supplemental data of [23]. The control cohort is from the NHLBI Women’s Health Initiative (WHI), which consists of 557 samples. The variant calls for each WHI sample are collected via the same pipeline with OV cases.

*RareProb-C* applied an exome-wide association analysis on the total of 7774 variants. It reports 9 genes harboring causal variants with significant *p*-values, which are *BRCA1*, *BRCA2*, *CHEK2*, *BRIP1*, *USP6*, *PALB2*, *ATM*, *PCSK7* and *FLT3*. Among these 9 genes, 5 genes (*BRCA1*, *BRCA2*, *CHEK2*, *BRIP1* and *USP6*) are highlighted as significant susceptibility genes associated to ovarian cancer in an integrated germline-somatic study on the same TCGA OV cases [23]. The association analysis results between *RareProb-C* and this research are shown in Table 3.

**Table 3:** Significant associated genes identified by *RareProb-C* comparing to the ones highlighted in the integrated germline-somatic research on the same dataset

Gene name	RareProb-C	OV research
BRCA1	3.0×10^−14^	2.0×10^−8^
BRCA2	4.5×10^−15^	8.9×10^−6^
CHEK2	2.4×10^−15^	0.11^∗^
BRIP1	5.2×10^−10^	0.11^∗^
USP6	3.3×10^−12^	Not Significant ^∗^
PALB2	5.2×10^−10^	Not Significant ^∗^
ATM	2.9×10^−9^	Not Significant
PCSK7	7.6×10^−8^	Not Significant
FLT3	5.8×10^−7^	Not Significant

^*^These 4 genes are considered to contribute to ovarian cancer susceptibility in the research, although without reaching the significance threshold (*P*<0.05)

Moreover, for the 3 genes (*ATM*, *PCSK7* and *FLT3*) that are not reported as significant in [23], our findings are also noteworthy. *ATM* is a known ovarian cancer associated gene reported in multiple researches [24, 25]. According to the literature review, we find that both *PCSK7* and *FLT3* are reported to be associated with ovarian cancer in [26], respectively. As a comparison, we also run *RWAS*+*SIFT*, *LRT*+*SIFT*, *RareCover* and *RareProb* on the same dataset. However, *RareCover* only identifies *BRCA1* and *BRCA2* with significant associations, while *RareProb* reports *BRCA2*, *CHEK2* and *YWHAE*. Unfortunately, we do not find literature supports *YWHAE*. Other approaches do not report significant results.

## Conclusions

In this article, we introduce an improved burden-test pipeline for cancer sequencing data, *RareProb-C*. This new pipeline is a model-free association approach. It considers the interactions among the given variants, by incorporating variant allelic frequencies and other estimations directly from sequencing data. It is able to overcome several known weaknesses of the existing collapsing methods. *RareProb-C* significantly extends and enhances the hidden Markov random field model in *RareProb* and technically estimates the hidden states and model parameter with fewer degrees of freedom. We apply *RareProb-C* to a set of TCGA ovarian cancer cases and a control cohort from NHLBI Women’s Health Initiative. *RareProb-C* successfully identifies several significant associations, which are strongly supported by multiple researches. In the comparisons of *RareProb-C* on simulation datasets under different simulation configurations, the results demonstrate that this new approach outperforms 6 popular approaches in terms of statistical power, sensitivity and specificity.
